# Propagation of distinct tau strains in neuronal systems with physiological tau expression

**DOI:** 10.1002/alz.090978

**Published:** 2025-01-03

**Authors:** Lenka Hromadkova, Kiley Urquhart, Chae Kim, Tracy Haldiman, Jiri Safar

**Affiliations:** ^1^ Case Western Reserve University, Cleveland, OH USA

## Abstract

**Background:**

Pathological tau forms from Alzheimer’s disease (AD) brains act as seeds, replicating in cells and forming tau aggregates in a template‐like manner. The exploration of this prion‐like pathogenic mechanism has predominantly occurred in transgenic mice and cell systems that overexpress tau protein and its truncated forms with pro‐aggregation mutations. However, these systems do not entirely capture the propagation kinetics and template conformational changes of various tau seeds. Our investigation focuses on tau propagation in cell systems with normal tau levels, emphasizing molecular conformation variability among tau strains, considering them as distinct populations in individual AD cases (Hromadkova et al., Cell Biosci., 2023).

**Method:**

We employed three types of cell systems: mouse primary neurons, differentiated SH‐SY5Y, and iPSCs‐derived neurons. We optimized culture conditions and timing for inoculation with AD‐tau isolates. The amount and conformational state of AD‐tau were characterized using conformational stability assay (CSA) and conformation‐dependent immunoassay (CDI). Subsequently, tau aggregates newly formed in neurons exposed to AD‐tau were subjected to immunostaining and cell lysis. We analyzed the results using confocal microscopy, immunoblot techniques, and CDI.

**Result:**

In all three systems, tau protein showed the tendency to aggregate when exposed to AD‐tau inoculates. During the early stages of inoculation, we noted the formation of tau aggregates with conformational properties resembling those found in the original AD‐tau isolates. Extended inoculation revealed increased aggregation, different from original AD‐tau, suggesting favorable propagation of highly seed‐competent tau entities in long‐term cultures.

**Conclusion:**

Our findings indicate that different tau strains manifest as dynamic entities, exhibiting variability not only in molecular conformations and structural arrangements but also in the kinetics of inter‐neuronal propagation. This diversity might contribute to the observed heterogeneity in AD phenotypes. The presence of different tau strains among AD patients introduces an added layer of complexity to disease progression and outcomes. Consequently, it may be worthwhile to consider more personalized therapeutic interventions tailored to individual patients.

